# Supplementary material for: A Multi-Point Identification Approach for the Recognition of Individual Leopards (Panthera pardus kotiya)
Source: Animals (Basel). 2022 Mar 6;12(5):660. doi: 10.3390/ani12050660 (PMC8909430; doi:10.3390/ani12050660)
Supplement: Supplementary file 1 [file animals-12-00660-s001.zip › Supplementary Figure S1.pdf]

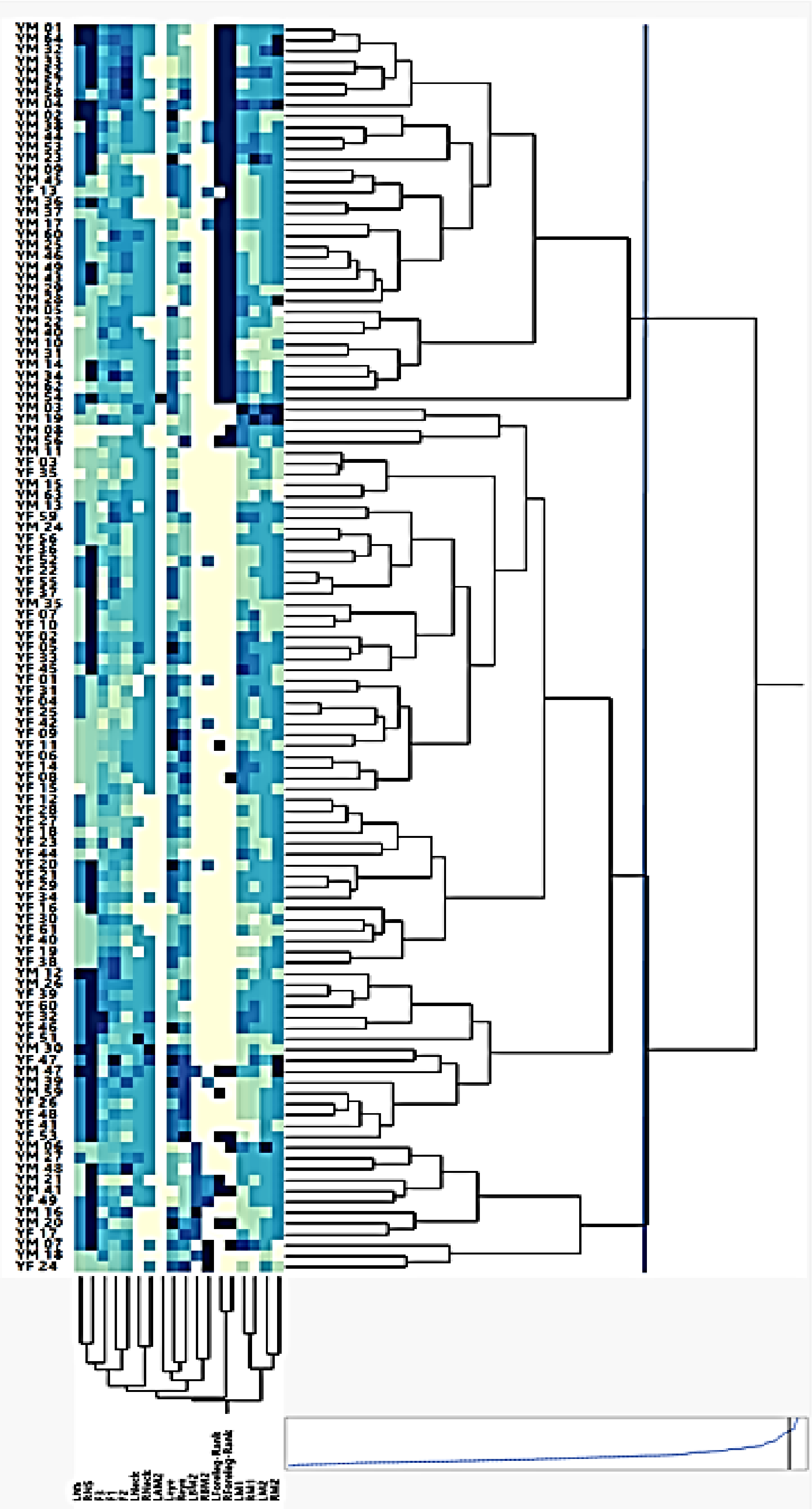

Supplementary Figure S1 Cluster analysis of 126 male and female leopards in Yala National Park according to their 17 spot distribution at areas around mystacial, nasal, eye and foreleg
