# Supplementary material for: A Multi-Point Identification Approach for the Recognition of Individual Leopards (Panthera pardus kotiya)
Source: Animals (Basel). 2022 Mar 6;12(5):660. doi: 10.3390/ani12050660 (PMC8909430; doi:10.3390/ani12050660)
Supplement: Supplementary file 1 [file animals-12-00660-s001.zip › Supplementary information S1.pdf]

Changes to spot patterns with an injury being documented.

Change in the spot marking of YM 7 - subsequent to the Documented Injury 1 of YM 7

The spot pattern of the Leopard coded as YM 7 was observed by the team since the year 2014. YM 7 was observed with a row of 3 spots just below the Mystacial Row 2, on the Right side of his face. YM 7 was recorded with an injury to the area of the rear end of the Muzzle towards the Left side of the face, in June 2018. The spot formation of the area was monitored on every sighting we had after the injury. The spot formation had changed after the injury, without returning to its original form. The Photos in Figure 6A shows the sequence of the YM 7 before the injury, the observation of the injury and the Photos after the injury. The spot formation of the Muzzle and side of the face of the area of the injury had changed from the original formation before the Injury and remains changed. This change is termed as a Rejig change. The Pattern of the Spot formation Marked by a circle in Figure 6A(a) has been changed as shown in Figure 6A(b), by the injury Figure 6A(c)

Change in spot markings of YM 16 – Subsequent to the Documented injury 1 of YM 16

The spot and Rosette formation of the Leopard YM 16 was observed by the team since the year 2017 (Figure 6B(a)). YM 16 was observed with an injury to the area just after his muzzle on the Right side of the face in October 2020 (Figure 6B(b)). The spot formation of the area was monitored on every sighting we had after the injury. The Spot formation of the area had changed after the injury, without returning to its original form (Figure 6B(c)). This change is termed as an Obliterate change as the spots in the area are not visible.

Change in spot markings of YM 16 – Subsequent to the Documented injury 2 of YM 16

The spot and Rosette formation of the Leopard YM 16 was observed by the team since the year 2017 (Figure 6C(a)). An Injury to the Upper area to the Right side of YM 16’s face was observed in February 2020 (Figure 6C(b)). The spot formation of the area was monitored on every sighting we had after the injury. The spot formation had changed after the injury without returning to its original form (Figure 6C(c)). This change is termed as an Obliterate change as the spots in the area have disappeared.

Change in spot markings of YM 27 – Subsequent to the Documented injury 1 of YM 27

The spot and Rosette formation of Leopard YM 27 was observed by the team since the year 2018 (Figure 6D(a)). An injury to an area of the Flank of YM 27 was observed in October 2020 (Figure 6D(b)). The spot and Rosette formation of the area was monitored on every sighting we had after the injury. The spot and Rosette formation had changed after the injury without returning to its

original form (Figure 6D(c)). This change is termed as an Rejig change as the Rosettes and spots in the area has changed its form.

Change in spot markings of YM 59 – Subsequent to the Documented injury 1 of YM 59

The spot and Rosette formation of Leopard YM 59 was observed by the team since July 2020 (Figure 6E(a)). An injury to the starting spot of the F2 spot formation, inner and parallel to the Right Genesis spot and the F3 segregation of the forehead of YM 59 was observed in January 2021 (Figure 6(b)). The spot and Rosette formation of the area was monitored on every sighting we had after the injury. The Spot parallel to the Genesis spot of the F2 Spot pattern formation of the Forehead has changed after the injury without returning to its original form Figure 6E(c)). The Spot which was present in the circled area in the Figure 6E(a) of November 2020 is less prominent and its shape has changed as shown in Figure 6E(c) after the Injury. This change is a Rejig Change.

Change in spot markings of YM 59 – Subsequent to the Documented injury 2 of YM 59

The spot and Rosette formation of Leopard YM 59 was observed by the team since July 2020 (Figure 6F(a)). An injury to the starting spot of the F2 spot formation, inner and parallel to the Right Genesis spot and the F3 segregation of the forehead of YM 59 was observed in January 2021 (Figure 6F(b)). The spot and Rosette formation of the area was monitored on every sighting we had after the injury. The spots of the inner side of the Left Genesis point in the F3 segregation of the Forehead has changed after the injury without returning to its original form. The spots of the F3 segregation have formed a deeper inward curvature than the original formation (Figure 6F(c)). This change is an Rejig Change as the formation of the F3 spots have changed.

Moreover, an injury at the F1 spot formation, observed in YM 3, YF 5, YM 2, YM 11 and YM 53. The spots in F1 segregation of the Forehead has changes after injury without returning to its original form.

Changes to spot patterns without an injury being documented.

Changes to the Spot Pattern Formation of YM 01 - Undocumented cause 01 of YM 01

The Leopard YM 01 has been observed by the team since the year 2004 (Figure 7A (c)). The Leopard was first observed as a Sub Adult. The spot and Rosette pattern formation of YM 01 was observed by the team for 13 years. The team had been photographing YM 01 before the inception of the formal data gathering process. The Spot formation of the area on the Right side of the Guide Arch on the side of the face has changed considerably without any injuries being documented (Figure 7A

(d)). This Change is an Rejig change as the spots of the area in concern is very much less prominent than the original form.

Changes to the Spot pattern Formation of YM 01 - Undocumented cause 02 of YM 01

The Leopard YM 01 has been observed by the team since the year 2004 (Figure 7B (c)). The Leopard was first observed as a Sub Adult. The spot and Rosette pattern formation of YM 01 was observed by the team for 13 years. The team had been photographing YM 01 before the inception of the formal data gathering process. The Spot formation of the area under the Right eye up to the start of the Muzzle on the Right side has changed considerably without any injuries being documented (Figure 7B(d)). This change is an Obliterate change as the prominence of the spot formations in the area described has diminished.

Changes to the Spot pattern Formation of YM 01 - Undocumented cause 03 of YM 01

The Leopard YM 01 has been observed by the team since the year 2004 (Figure 7C (c)). The Leopard was first observed as a Sub Adult. The spot and Rosette pattern formation of YM 01 was observed by the team for 13 years. The team had been photographing YM 01 before the inception of the formal data gathering process. The Spot formation of the Right side Mystacial Row 1 has changed considerably without any injuries being documented. The Right side Mystacial Row 1 spot count in the year 2004 was counted as 3 spots. The prominent visibility of all 3 spots were the same. The Prominence of a Mystacial spot has diminished by the year 2016. Only 2 prominent spots are visible (Figure 7C (b)). This change is a Rejig Change as the prominence of the Mystacial has diminished.

Changes to the Spot Pattern Formation of YM 01 - Undocumented cause 04 of YM 01

The Leopard YM 01 has been observed by the team since 2004 (Figure 7D (c)). The Leopard was first observed as a Sub Adult. The spot and Rosette pattern formation of YM 01 was observed by the team for 13 years. The team had been photographing YM 01 before the inception of the formal data gathering process. The Spot formation of the F1 and F2 segregations of the Forehead had changed considerably by the year 2016 without any injuries being documented (Figure 7D (d)). This Change is an Obliterate change as some of the F1 and F2 spots of the Forehead have diminished.

Changes to the Spot pattern Formation of YF 03 - Undocumented cause 01 of YF 03

The Leopard YF 03 was observed by the team since the year 2010 (Figure 7E (c)). This Leopard was first observed as a sub adult. The team has been observing the spot and rosette formation of YF 03 for 6 years. The Spot formation of the Left side Mystacial Row 1 has changed considerably without any injuries being documented. The Left side Mystacial Row 1 spot count in the year 2010 was counted as 2 spots. The prominent visibility of both spots was the same. The Prominence of both Mystacial spots have diminished by the year 2015 (Figure 7E (d)). This change is an Obliterate Change as the prominence of a Mystacial has disappeared.

Changes to the Spot pattern Formation of YF 01 - Undocumented cause 01 of YF 01

The Leopard YF 01 was observed by the team since the year 2014 (Figure 7F (c)) . This Leopard was first observed as an Adult. The team has been observing the spot

and rosette formation of YF 01 for 6 years. The Spot formation of the Right side F1 Segregation has changed considerably without any injuries being documented. The Prominence of the spots in the Right side F2 have diminished by the year 2019 (Figure 7F (d)). This change is a Rejig Change as the prominence of the Mystacial spot has diminished.

Changes to the Spot pattern Formation of YM 07- Undocumented cause 01 of YM 07

The spot pattern of the Leopard coded as YM 07 was observed by the team since the year 2014 (Figure 7G (c)). The F1 Spot formation of the forehead of YM 07 was visually Prominent in our observations from the first observation and subsequent observations spanning up to 2021. We observed a change in the F1 Spot formation of YM 07 in 2021. This change is termed as a Obliterate change (Figure 7G (d)). This change is an Obliterate change as some spots have disappeared . The prominence of the spots that are visible in the F1 area have diminished. The diminishing of the prominence of those spots amount to a Rejig Change.

Changes to the spot pattern of YM 32 - Undocumented cause 01 of YM 32

The Spot pattern of the Leopard YM 32 was observed by us since the year 2018 (Figure 7H (c)). The F1 and F2 spot formations of the forehead were prominent in our observations up to January 2021 We observed a change in the F1 and F2 spot formation of YM 32 in October 2021. A prominent spot marking of the F1 spot formation and a prominent spot marking of the F2 spot formation is not visible (Figure 7H (d)). An Obliterate change had occurred in both the F1 and F2 spot formations of YM 32.

Moreover, during the presence absence survey, several other changes were observed in Forehead area (F1, F2, F3) in YM3, YF 3, YF 6, YF 20, YF 24 and YM 53. In most of the cases the prominence has reduced except in YM 3 the spot pattern/shape has changed. Accordingly, spot variations can occur even without an injury.
