# Supplementary material for: A Multi-Point Identification Approach for the Recognition of Individual Leopards (Panthera pardus kotiya)
Source: Animals (Basel). 2022 Mar 6;12(5):660. doi: 10.3390/ani12050660 (PMC8909430; doi:10.3390/ani12050660)
Supplement: Supplementary file 1 [file animals-12-00660-s001.zip › Supplementary information S2.pdf]

Errors in Visual Observation of sex

An observation was made by us on a sudden change that can happen in the physical body of a Leopard. We had identified YM 52, a Sub adult male Leopard in 2020. During one of the sightings, we observed that his testicles, which were clearly visible on all other occasions, were not visible at all, making the Leopards anatomical structure change, as shown in Figure 10 (A,B,C),. This change was observed for 10 seconds and the testicles resurfaced into the normal position as shown in Figure 10D. A photo of a similar aged Male Leopard, YM 43, is presented in Figure 7E to make evident the common form of the Testicles when running. This change has not been documented in a Leopard's anatomy in the past.

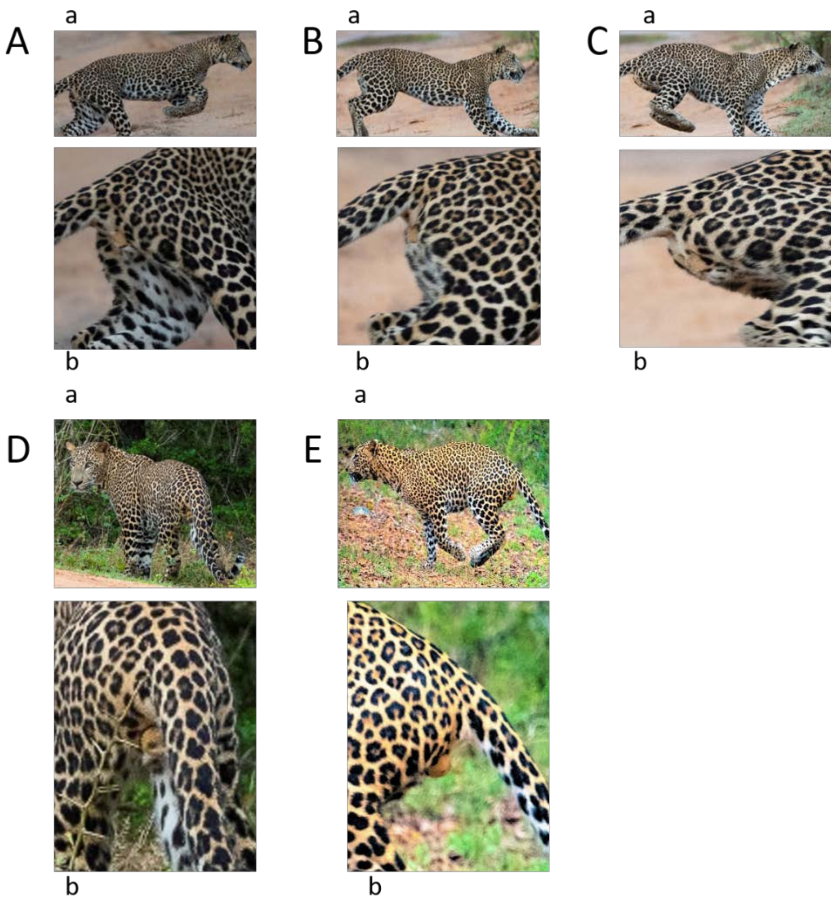

Supplementary information S2: Errors in visual observation of Sex

(A), (B), (C) Visual identification of YM 52, testicles were not visible at running position (D) YM 53 testicles were resurfaced to normal position (E) YM 43 Common form of testicles when running
