# Supplementary material for: A Multi-Point Identification Approach for the Recognition of Individual Leopards (Panthera pardus kotiya)
Source: Animals (Basel). 2022 Mar 6;12(5):660. doi: 10.3390/ani12050660 (PMC8909430; doi:10.3390/ani12050660)
Supplement: Supplementary file 1 [file animals-12-00660-s001.zip › Supplementary Table S1.pdf]

Table S1: Principal Component Analysis (PCA) Factor loads for every variable in the seven main clusters Eigen- values for the seven obtained components. The percentage of the variance explained by each axis is also indicated. Strong Interactions in the main three clusters are in bold.

	Prin1	Prin2	Prin3	Prin4	Prin5	Prin6	Prin7	Prin8
LNS	**0.2914**	**0.26131**	0.15393	0.29613	-0.00776	0.06439	-0.05826	0.06921
LM1	0.11735	0.19983	-0.51919	-0.07557	0.18098	0.00953	0.33536	0.01248
LM2	0.22842	0.14381	-0.30789	0.17239	0.06118	-0.12142	-0.21923	0.0283
LAM2	0.0725	0.02792	0.07946	-0.11129	-0.03783	-0.11885	0.14384	0.88693
LBM2	0.03681	0.01818	0.18168	0.10403	0.51882	0.07736	-0.32926	-0.0777
RNS	0.20542	**0.27855**	**0.27065**	0.3865	0.07813	0.13338	0.08093	0.12319
RM1	0.17101	**0.27187**	-0.45569	0.09835	0.09523	0.11203	0.28582	-0.15112
RM2	0.19145	**0.28375**	-0.12664	0.06468	-0.23761	0.05953	-0.43736	0.07552
RAM2	-0.04873	-0.17568	0.14164	0.14981	-0.30817	-0.01366	0.35868	-0.22703
RBM2	-0.01424	-0.18603	0.1267	0.13129	0.60865	0.19808	0.12338	-0.00448
F1	0.15515	**0.31515**	**0.27384**	-0.11069	-0.28534	0.13453	-0.06765	-0.219
F2	0.21566	**0.26363**	**0.2228**	-0.47157	0.15973	0.03599	0.03182	-0.15989
F3	0.04716	**0.22291**	**0.29523**	0.09196	-0.01659	0.13249	0.50749	0.01594
Leye	**0.33624**	-0.2707	0.01733	0.27758	0.00464	-0.32572	0.04045	-0.01767
LNeck	**0.36412**	-0.18703	0.04408	0.10063	-0.02303	-0.40737	0.03212	-0.13776
LForeleg_Rank	**0.38116**	-0.0701	0.12744	-0.28648	0.1045	-0.37499	0.07526	-0.07358
Reye	**0.26749**	-0.34297	-0.05169	0.06338	-0.12273	0.48976	-0.09374	0.0656
RNeck	**0.31783**	-0.32759	-0.07818	0.04364	-0.14223	0.37847	0.05753	0.00464
RForeleg_Rank	**0.30914**	-0.12599	-0.02265	-0.48218	0.04247	0.23428	-0.00206	0.05902
Eigenvalue	3.2663	2.0764	1.8625	1.3554	1.3146	1.2403	1.1453	1.0141
Percent (%)	17.191	10.928	9.803	7.134	6.919	6.528	6.028	5.338
Cum Percent	17.191	28.12	37.922	45.056	51.975	58.503	64.531	69.868
