# Supplementary material for: A Multi-Point Identification Approach for the Recognition of Individual Leopards (Panthera pardus kotiya)
Source: Animals (Basel). 2022 Mar 6;12(5):660. doi: 10.3390/ani12050660 (PMC8909430; doi:10.3390/ani12050660)
Supplement: Supplementary file 1 [file animals-12-00660-s001.zip › Supplementary Table S2.pdf]

Table S2: **Data set for Injuries of Leopards -**

Obliterate changes (Completely dissapeared markings)					
Method - Count					
ID Code	Injury No	Injury Area	Reference photo	Number of spots at Capture	Number of spots at recapture
YM 16	D 1	Near Muzzle (R)	5 B	10	0
YM 16	D 2	Upper side of face (L)	5 C	4	0
YM 01	U 2	Under the eye ®	6 B	7	0
YM 01	U 4	Forehead (F1)	6 D	15	7
YF 03	U 1	Mystacial spots (L)	6 E	2	0
YM 07	U 1	Forehead (F1)	6 G	10	4
YM 32	U 1	Forehead (F1) (F2)	6 H	15	10
YM 3	U1	Forehed F1		11	7
YF 5	U1	Forehead F1		15	12
YM 2	U1	Forehead (F1)		18	9
YM 11	U1	Forehead (F1)		14	10
YM 53	U1	Forehead (F1)		12	10

Rejig changes (Markings where the formation has changed)				
Method - Presence absence survey				
ID Code	Injury No	Injury Area	Reference photo	Rank of change in spot or rosette pattern formation at recaptur
YM 07	D 1	Below Mystacial area M2 (R)	5A	2
YM 27	D 1	Flank (L)	5D	2
YM 59	D 1	Forehead (F2)	5E	2
YM 59	D 2	Forehead (F3)	5F	2
YM 01	U 1	Side of face (Right of Guide arch(R))	6A	1
YM 01	U 3	Mystacial area (R)	6C	1
YF 01	U 1	Forehead (F1)	6F	1
YM 3	U2	Forehead (F2 & F3)		2
YF 3	U2	Forehead (F1)		1
YF 6	U1	Forehead (F1)		1
YF 20	U1	Forehead (F1 & F3)		1
YF 24	U1	Forehead (F1)		1
YM 53	U2	Forehead (F2)		1

Ranking guide for Rejig changes
Rank 1 Prominance reduced
Rank 2 Prominence has reduced
